# Factors that contribute to trait mindfulness level among hospitalized patients with major depressive disorder

**DOI:** 10.3389/fpsyt.2023.1144989

**Published:** 2023-06-29

**Authors:** Cai-Fang Ji, Guan-Hui Wu, Xiang Dong Du, Gui-Xian Wang, Li-Li Liu, Mei-E. Niu, Robert Logan, Fan-Zhen Kong

**Affiliations:** ^1^Department of Psychology, Suzhou Guangji Hospital, The Affiliated Guangji Hospital of Soochow University, Suzhou, China; ^2^Department of Neurology, The Affiliated Suzhou Hospital of Nanjing Medical University, Suzhou Municipal Hospital, Suzhou, China; ^3^Moral Education Research Center, Tsinghua University, Beijing, China; ^4^Department of Nursing, The First Affiliated Hospital of Soochow University, Suzhou, China; ^5^Department of Biology, Eastern Nazarene College, Quincy, MA, United States; ^6^Department of Nursing, Suzhou Guangji Hospital, The Affiliated Guangji Hospital of Soochow University, Suzhou, China

**Keywords:** mindfulness, depression, major depression disorder, trait mindfulness, FFMQ

## Abstract

Mindfulness training among patients with major depressive disorder (MDD) reduces symptoms, prevents relapse, improves prognosis, and is more efficient for those with a high level of trait mindfulness. Upon hospital admission, 126 MDD patients completed the Beck Depression Inventory (BDI), World Health Organization Quality of Life Brief, Five-Factor Mindfulness Questionnaire (FFMQ), and the Rumination Response Scale (RRS). The 65 patients that scored less than the median of all subjects on the FFMQ were placed into the low mindfulness level (LML) group. The other 61 patients were placed in the high mindfulness level (HML) group. All facet scores were statistically different between the mental health assessment scores of the HML and LML groups except for RRS brooding and FFMQ nonjudgement. Trait mindfulness level exhibited a negative and bidirectional association with MDD severity primarily through the facets of description and aware actions. Trait mindfulness was also related positively with age primarily through the facets of nonreactivity and nonjudgement. Being married is positively associated with trait mindfulness levels primarily through the facet of observation and by an associated increase in perceived quality of life. Mindfulness training prior to MDD diagnosis also associates positively with trait mindfulness level. Hospitalized MDD patients should have their trait mindfulness levels characterized to predict treatment efficiency, help establish a prognosis, and identify mindfulness-related therapeutic targets.

## Introduction

Major depressive disorder (MDD), also known as clinical depression, is a common and debilitating mood disorder. Risk for MDD increases with age. About 6% of adults worldwide have MDD (1). However, it is estimated that up to 13.3% of the world’s elderly population has MDD (2). To receive a diagnosis, individuals must have at least five recognized symptoms that persist for two weeks or more. Examples of recognized symptoms include diminished mood, reduced interest, or pleasure in almost all activities that once were pleasurable and of interest, reduction in physical movement, increased fatigue, reduced ability to concentrate and make decisions, recurrent thoughts of death or suicidal ideation, and changes in sleep and appetite (3, 4). A biological association between advanced age, MDD, suicidal risk, and diminished clinical outcomes is a pathological upregulation of specific inflammatory cytokines (5). The family and societal burden of MDD ranks first among all neuropsychiatric diseases and is a leading cause of disease burden worldwide (6). Tragically, MDD has a low rate of clinical recognition, medical consultation, and effective treatment (6, 7).

In the cases of unsuccessful therapeutic intervention, it behooves the clinician to pursue novel alternatives. For example, buprenorphine has been effective at treating patients who are otherwise resistant to traditional pharmacological interventions (8). Nonpharmacological interventions are also critical to incorporate into the treatment plan for MDD patients, such as psychotherapy. Mindfulness-related psychotherapy has become a prevalent form of modern psychological treatment for MDD and a variety of other disorders. Mindfulness is the meditative process of shifting attention from the perceived activity of the mind to the perception of present experiences in a nonjudgmental and relaxed state (9–11). Stated more simply, mindfulness meditation is “paying attention in a particular way: on purpose, in the present moment and nonjudgmentally” (12). The nonjudgmental aspect of mindfulness allows for the acknowledgement and acceptance of mental activities, such as patterns of attention and sensory processing.

The capacity for mindfulness is an innate and relatively stable human trait that can be improved with intervention (13). Mindfulness training has been shown to help patients improve their emotional regulation and diminish the occurrence of habitual automatic negative thought patterns, such as depression-related rumination and catastrophizing (11, 14). Importantly, mindfulness training helps patients with MDD to prevent relapses and improves their prognosis (9–11, 15). However, trait mindfulness level impacts the magnitude of the effect that mindfulness training has (16). Indeed, scored trait mindfulness dimensions can reliably predict the extent that most of the assayed dimensions can change due to mindfulness-based interventions (17).

Patients with MDD have on average significantly lower mindfulness levels compared to healthy controls on scored tests such as the Mindful Attention Awareness Scale (18). The Five-Facet Mindfulness Questionnaire (FFMQ) is a popular and well-established tool for surveying the robustness of a person’s mindfulness (19). The FFMQ addresses a person’s capacity to observe their environment and inner experience, a person’s capacity to describe their observations, the depth of awareness a person has of their actions, a person’s capacity to accept their emotions for what they are and without judgment, and finally, a person’s ability to perceive their emotions without needing to react to them. The FFMQ is used in this study to score trait mindfulness levels among MDD patients.

MDD is a prevalent mood disorder that significantly impairs a person’s ability to engage in life with enjoyment, vigor, and cognitive acuity. Mindfulness-based psychotherapy has become widely incorporated into the treatment of many affective and behavioral disorders, including MDD. Trait mindfulness, also referred to as dispositional or baseline mindfulness, influences the effectiveness of mindfulness-based interventions (20). We hypothesize that the trait mindfulness levels among patients hospitalized for MDD will significantly differ according to a characteristic profile of factors. Therefore, in this current study we investigated the trait mindfulness levels of patients hospitalized for MDD and the factors that influence their trait mindfulness at baseline. The findings of this study can help clinicians identify MDD patients who would benefit the most from mindfulness-based interventions and aid in the development of a prognosis.

### Subjects

The subjects were admitted to Suzhou Guangji Hospital in Suzhou China, from July 2015 to June 2016 for MDD. The study inclusion criteria required that subjects met the diagnostic criteria for DSM-V major depressive disorder, had a total BDI score ≥ 5 at the time of enrollment, were aged 18–65 years, and a signed informed consent from either the patients or their family members. The study exclusion criteria included a history of manic episodes, suicide attempts, psychoactive drug dependence, a mental disorder caused by a disease other than MDD, and any other physical disease that is active and severe. The study was approved by the Suzhou Guangji Hospital Ethics Committee. The fact that the data were collected six years ago is not considered a limitation of this study. The findings remain relevant because the lifestyle of those who live in China hardly change over the years. Now that the transient and localized COVID-19 related social distancing measures have largely been dissolved, these results are reinstated as critical to explore and understand.

## Methods

### Assessment tools

We employed several validated self-report questionnaires to quantify various dimensions of mindfulness. For example, we relied on the Beck Depression Inventory (BDI) to score the severity of physical and cognitive symptoms related to depression (21). The BDI and the more modern BDI-II consists of 21 questions and is designed for patients 13 years old and older. The BDI and BDI-II tools have strong evidence of convergent and discriminatory validation across many clinical and non-clinical sample populations (22). In this study, the BDI test was used, as is common in Chinese hospitals.

We also used the World Health Organization Quality-of-Life BREF (WHOQOL-BREF) to score a patient’s quality of life across four dimensions: physical health, psychological health, health of social relationships, and how supportive the patient’s environment is in promoting health (23). The WHOQOL-BREF, which consists of 26 questions, was developed as an abbreviated version of the WHOQOL-100, which contains 100 questions. The WHOQOL-BREF has shown strong relation with the WHOQOL-100 and demonstrates good convergent and discriminatory validation (24).

We used the Five-Facet Mindfulness Questionnaire (FFMQ) to score the level of a patient’s underlying factors of mindfulness across 39 questions. These facets include observation, description, aware actions, non-judgmental inner experience, and non-reactivity (19). The FFMQ has been used in hundreds of studies to assess mindfulness and has been extensively validated. However, there has been some recent debate about its discriminant validity across cultures (25, 26).

Finally, we used the 22-question Rumination Response Scale (RRS) to score a patient’s depression symptoms and response to their depression by assessing their tendencies toward two opposite subtypes of rumination: brooding and reflection (27). Reflection seeks to establish a cognitive explanation and solution for depressed feelings, and thus, necessitates the enhancement of self-awareness and situational insight. Brooding is a maladaptive and passive rumination that is focused on how current or past circumstances do not align with a preferred situational outcome. The RRS was originally proposed in 1991 and was modified in 2003 (28–30). The RRS has been validated for various cultures, such as Turkish, French, Spanish, and Chinese cohorts (31–34).

### Test reliability and validity

The Cronbach’s α coefficient and the half-score reliability coefficient using the Spearman-Brown formula were used to analyze internal test reliability. The Kaiser-Meyer-Olkin (KMO) Test was used to assess sampling adequacy and the appropriateness of the data for factor analysis. The BDI, FFMQ, WHOQOL-BREF, and RRS scales showed good reliability and validity in this study. The lowest Cronbach’s α coefficient values for BDI, FFMQ, WHOQOL-BREF, and RRS were 0.871, 0.756, 0.897, and 0.851, respectively. The lowest Spearman-Brown coefficient values for BDI, FFMQ, WHOQOL-BREF, and RRS were 0.763, 0.913, 0.846, and 0.986, respectively. Therefore, the Cronbach’s α and the Spearman-Brown coefficient values were above 75.6% and achieved high reliability. The lowest Kaiser-Meyer-Olkin (KMO) values for the BDI, FFMQ, WHOQOL-BREF, and RRS assessments were 0.859, 0.749, 0.844, and 0.911, respectively. The statistical significance of Bartlett’s sphericity test for all assessments reached *p* < 0.01. Therefore, both the KMO and Bartlett’s sphericity test values were satisfactory. The cumulative variance contribution rate of each scale ranged from 48.761 to 66.638%, indicating that the content of each scale in this study meets the requirements necessary to explain the evaluated information. The cumulative variance contribution rate was the lowest with the FFMQ, which was 48.761%. The results of test reliability and validity confirmation of BDI, FFMQ, WHOQOL-BREF, and RRS are presented in Table 1.

**Table 1:** Test reliability and validity of BDI, FFMQ, WHOQOL-BREF, and RRS.

	*α*	*r_full_*	KMO	Approx. *χ*^2^	df	CumVar
BDI	0.871	0.763	0.859	601.141	78	66.638
FFMQ	0.756	0.913	0.749	2278.987	741	48.761
WHOQOL	0.897	0.846	0.844	181.619	276	51.648
RRS	0.851	0.986	0.911	1643.178	231	59.162

α, Cronbach’s alpha; r_ful_, Spearman-Brown coefficient; KMO, Kaiser-Meyer-Olkin value; Approx *χ*^2^, Bartlett’s Test of Sphericity approximate chi-square value; df, Bartlett’s Test of Sphericity degrees of freedom (all measures of Bartlett’s Test of Sphericity were statistically significant at *p* < 0.001); CumVar, cumulative variance contribution rate (%), BDI, Beck Depression Inventory; FFMQ, Five-Factor Mindfulness Questionnaire; WHOQOL, World Health Organization Quality of Life BREF; RRS, Rumination Response Scale.

### Demographic classifications

Patient demographic and clinical data were collected including age, gender, religious belief, education level, marital status, income level, admission date and time, course of disease, and medication. Education and income level were assessed bimodally. Education level was either recorded as “high” if it exceeded beyond 12 years, otherwise it was recorded as “low.” Income level was recorded as either “high” if the *per capita* month household income exceeded $500 USD, otherwise it was recorded as “low.” This bimodal assignment approach preserved our ability to perform a reliable statistical assessment. Dividing the patient data into multiple tiers reduced the per-group sample size n and so only two tiers were used. Assessment of patient FFMQ performance was also binary. If a patient earned a FFMQ score of greater than the median score of 96, the patient was recorded as having a “high” mindfulness level (HML), otherwise the patient would be considered to have a “low” mindfulness level (LML). Other studies have adopted a bimodal high and low classification of subject scores on the FFMQ according to the subject’s score relative to the mean of all scores (35).

### Statistical analysis

Statistical analysis was performed using SPSS 22.0 software. Quantitative results that exhibited normal distribution are presented as the mean ± standard deviation (*x-bar* ± *SD*), as calculated by the independent sample *t*-test. Quantitative results that did not exhibit normal distribution are presented as the independent sample median, as calculated by the nonparametric Mann–Whitney U test. The count data were analyzed by chi-square test and expressed as the number of cases (percentage). The independent factors that contribute to mindfulness level were analyzed by binary logistic regression analysis and linear regression analysis. Statistical difference between groups was determined to be at *p* < 0.05.

## Results

### Demographic characteristics of patients with MDD

A total of 126 patients that met the inclusion criteria were enrolled in the study, consisting of 58 males and 68 females, aged 41.40 ± 14.29 years. Although the enrollment criteria required that patients have a clinical diagnosed case of DSM-V MDD, no patient had a mild case. All enrolled MDD patients had either a moderate or severe classification. In addition, the enrollment criteria required a BDI score of ≥5, but all patients scored >8. The range and interpretation of BDI scores include a “mild” result between scores 5 and 7, a “moderate” ranking between 8 and 15, and any score above 15 indicates “severe.” There were 12 patients with a BDI score of 8 < 15 and 114 patients with BDI score of >15. The LML group consisted of 65 patients who scored below the median FFMQ score of 96. The HML group consisted of 61 patients with an FFMQ score above 96. Compared to the LML group, the patients in the HML group were older (*t* = −2.107, *p* = 0.037). However, there were no differences (*p* > 0.05) between the LML and HML groups in gender, number of hospital admissions, religious belief, course of disease, education level, marital status, income level and medication use. The MDD patient demographics are presented in Table 2 according to their mindfulness level assigned at hospital admission.

**Table 2:** Demographic characteristics of patients hospitalized with major depressive disorder according to their trait mindfulness.

	LML (*n* = 65)	HML (*n* = 61)	*t*-score	*p*-value
Age (years)	38.83 ± 14.86 4	4.15 ± 13.36	−2.107	0.037
Sex			0.108	0.742
Male	29 (44.6%)	29 (47.5%)		
Female	36 (55.4%)	32 (52.5%)		
Number of hospitalizations			0.526	0.468
≤ 2	43 (66.2%)	44 (72.1%)		
>2	22 (33.8%)	17 (27.9%)		
Religious			0.004	0.95
No	53 (81.5%)	50 (82.0%)		
Yes	12 (18.5%)	11 (18.0%)		
Course of disease (days)	28 (6.5, 90)	36 (7.5, 96)	−0.636	0.525
Education			0.495	0.482
Low	23 (35.4%)	18 (29.5%)		
High	42 (64.6%)	43 (70.5%)		
Marriage Status			1.661	0.198
Married	41 (63.1%)	45 (73.8%)		
Unmarried	24 (36.9%)	16 (26.2%)		
Income			0.955	0.329
Low	36 (55.4%)	39 (63.9%)		
High	29 (44.6%)	22 (36.1%)		
Medication			1.354	0.245
No	11 (16.9%)	6 (9.8%)		
Yes	54 (83.1%)	55 (90.2%)		

LML, low mindfulness level; HML, high mindfulness level.

### Comparison of BDI, FFMQ, WHOQOL-BREF, and RRS component scores between mindfulness groups

Unsurprisingly, patients with MDD in the HML group scored better on measurements of mindfulness, depression, response to a depressed mood, and quality of life than the patients with MDD in the LML group. The BDI score of MDD patients in the HML group was significantly lower than the BDI score of patients with MDD in the LML group (*z* = −4.559, *p* < 0.001). For four out of the five facets of mindfulness measured by the FFMQ (observation, description, mindful actions, and nonreactivity), the MDD patients in the HML group scored significantly higher than those in the LML group (*p* < 0.01). However, there was no statistical difference in the nonjudgmental inner experience metric between the two groups (*t* = 0.360, *p* = 0.719). MMD patients in the HML group scored significantly better on the WHOQOL-BREF than the LML group (*p* < 0.01). Consistent with these findings, patients with MDD in the HML group had RRS scores that were statistically lower than the MDD patients in the LML group for symptom rumination and reflective pondering (*p* < 0.01). However, there was no significant difference between the HML and LML group scores for the RRS brooding metric (*z* = −1.865, *p* = 0.062). The performance and statistical analysis of the HML and LML groups of patients with MDD on the BDI, FFMQ, WHOQOL-BREF, and RRS test components are presented in Table 3.

**Table 3:** Depression, mindfulness, quality of life, and rumination quantification among patients with major depressive disorder according to their mindfulness groups.

	LML	HML	*t*-score	*p*-value
BDI	27 (23, 31)	20 (17, 27)	−4.559	0.000
*FFMQ*				
Observation	20 (16, 25)	24 (21, 28)	−3.012	0.003
Description	20 (16.5, 22)	23 (26, 31)	−7.235	0.000
Act with awareness	12 (10, 18)	21 (17, 25.5)	−5.895	0.000
Nonjudgement	14.48 ± 5.72	14.82 ± 4.89	−0.36	0.719
Nonreactivity	19.02 ± 4.91	21.21 ± 4.21	−2.689	0.008
Total	89 (82.5, 94)	104 (101, 116.5)	−9.683	0.000
*WHOQOL-BREF*				
Physical health	10.29 (9.71, 12.29)	12.57 (10.57, 13.71)	−3.475	0.001
Psychological health	10.67 (9.33, 11.33)	12 (10.67, 13.33)	−4.868	0.000
Social relationships	10.67 (9.33, 11.33)	13.33 (12, 14.67)	−3.981	0.000
Environment	12.55 ± 1.88	13.56 ± 2.21	−2.772	0.006
Total	73.17 ± 9.24	82.07 ± 10.56	−5.04	0.000
*RRS*				
Rumination	25.78 ± 5.79	21.66 ± 6.66	3.72	0.000
Reflective pondering	14 (12, 16)	11 (9, 14)	−3.751	0.000
Brooding	11 (8, 11)	8 (7, 11)	−1.865	0.062
Total	49.4 ± 10.54	42.16 ± 12.11	3.583	0.000

LML, low mindfulness level; HML, high mindfulness level; BDI, Beck Depression Inventory; FFMQ, Five-Facet Mindfulness Questionnaire; WHOQOL-BREF, World Health Organization Quality-of-Life BREF; RRS, Rumination Response Scale. The nested subcategories represent the various facets that are assayed under the assessment tool named in boldened text.

### Logistic regression analysis of factors that contribute to trait mindfulness level for patients with MDD

The dependent variable was the dichotomous grouping of mindfulness level. The independent variables were the factors that contributed to trait mindfulness, analyzed by univariate analysis with *p* < 0.2. Binary logistic regression analysis using the stepwise method showed that BDI score (Exp(*B*) 0.914, 95% CI 0.840–0.994, *p* = 0.035) was significantly negatively associated with mindfulness level, while the psychological health domain of the WHOQOL-BREF instrument was significantly and positively associated with mindfulness level (Exp(*B*) 1.458, 95% CI 1.110–1.915, *p* = 0.007). Additionally, the age of the subjects revealed significant logistic regression with trait mindfulness (Exp(*B*) 1.031, 95% CI 1.000–1.062, *p* = 0.048). The results of the binary logistic regression analysis of factors that contribute to trait mindfulness level are shown in Table 4.

**Table 4:** Logistic regression analysis of factors that affect trait mindfulness among patients with major depressive disorder.

	*B*	*p*	Exp(*B*)	95% CI
BDI scores	−0.09	0.035	0.914	0.840–0.994
WHOQOL-BREF Psychological health domain scores	0.377	0.007	1.458	1.110–1.915
Age	0.03	0.048	1.031	1.000–1.062

*B*, The set of coefficients estimated for the model; *p*, the level of statistical significance; Exp(*B*), *e* raised to the value of the regression coefficient. *e* is Euler’s number. Exp(*B*) represents the odds of an event change. Cl, confidence interval.

### Multiple linear regression analysis of factors that contribute to trait mindfulness level and related metrics for patients with MDD

The dependent variables were the FFMQ metric scores. The independent variable were all other metrics analyzed by univariate analysis with *p* < 0.2. Multiple stepwise linear regression analysis showed that FFMQ scores were positively related with age and marital status, and negatively related with BDI score. The analysis of each metric showed that observation was positively associated with marital status, nonreactivity was positively associated with age, description and acting with awareness were negatively associated with BDI score, and nonjudgment was positively associated with age and reflective pondering. The results of multiple linear regression analysis of factors that contribute to of trait mindfulness level and each metric are shown in Table 5.

**Table 5:** Multiple linear regression analysis of the five factors that affect trait mindfulness among patients with major depressive disorder.

	*B*	𝜷	*p*-value	95% CI
*Total FFMQ score*				
Age	0.185	0.199	0.024	0.025–0.345
Marital status	4.873	0.171	0.047	0.067–9.678
BDI score	−0.633	−0.299	0.0012	−1.125 – −0.141
*FFMQ observation*				
Marital status	4.508	0.331	0.001	1.852–7.164
*FFMQ nonreactivity*				
Age	0.078	0.239	0.023	0.011–0.145
*FFMQ description*				
BDI score	−0.401	−0.421	0.001	−0.641 – −0.162
*FFMQ act with awareness*				
BDI score	−0.442	−0.384	0.003	−0.697 – −0.147
*FFMQ nonjudgment*				
Age	−0.078	−0.211	0.028	−0.148 – −0.009
Reflective pondering	−0.913	−0.592	0.006	−1.544 – −0.272

*B*, the unstandardized regression coefficient; 𝜷, the standardized regression coefficient; FFMQ, Five-Facet Mindfulness Questionnaire.

## Discussion

**Figure 1:**
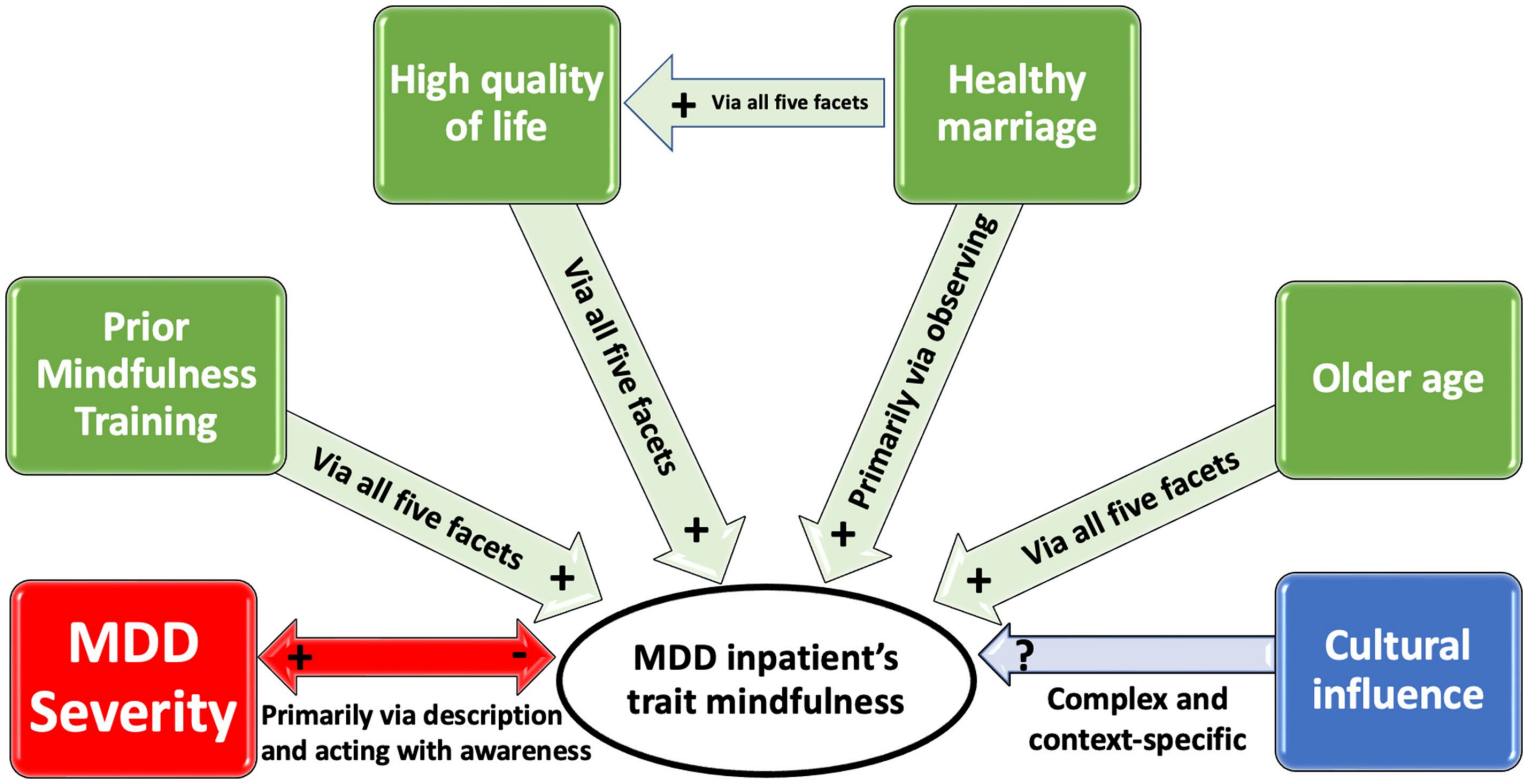
Six factors have a positive, negative or context-specific effect on trait mindfulness of inpatients with MDD in China and their mechanisms of impact. Red and (+, −) represents a negative inverse and bidirectional correlation. Green and (+) represents a positive correlation. Blue and (?) represents a complex and context-specific correlation. “Via all five facets” refers to the mindfulness facets as scored on the FFMQ.

Our study has demonstrated that MDD severity, mindfulness training prior to MDD diagnosis, general psychological health related to quality of life, being married, and advanced age are all independent factors, capable of influencing trait mindfulness level, as shown in Figure 1. This study has revealed an inverse relation between MDD severity, as reported by BDI score, and the baseline level of mindfulness. Other studies have corroborated the independent negative association between trait mindfulness and severity of MDD symptoms. For example, adult patients with severe MDD and a history of attempted suicide have a low level of mindfulness (36). Furthermore, patients with primary Generalized Anxiety Disorder (GAD) and co-morbid MDD have been shown to have statistically lower levels of mindfulness compared to patients with only GAD (37). This study has also identified a negative relation between scores on the FFMQ facets of mindful description and acting with awareness with the severity of MDD as reported by BDI score. A similar finding of another study has shown that the FFMQ facet scores of nonjudgement and nonreactivity negatively relate to the depression-linked symptoms of distress and anhedonia (38). Therefore, MDD severity measured by the BDI is an independently contributing factor to trait mindfulness level. Conversely, the level of mindfulness measured by the FFMQ negatively predicts MDD. It seems likely that the five aspects of the FFMQ could represent targeted areas of intervention to either treat or prevent MDD.

Mindfulness-based interventions are effective at mitigating the symptoms of severe MDD, reducing the risk of MDD relapse, and improving low levels of mindfulness among those with subclinical symptoms of mental health conditions (39). The greater the baseline severity of MDD, or the lower the level of mindfulness among MDD patients prior to engaging in mindfulness-based intervention, the greater the treatment efficiency, even in a nonclinical setting (16, 40, 41). However, regardless of the efficiency of treatment effect, trait mindfulness levels among MDD patients can be positively influenced by mindfulness-based training prior to a diagnosis and throughout treatment.

We have demonstrated that there is a positive relation between the psychological health domain score of the WHOQOL-BREF scale and trait mindfulness among MDD patients. The findings of many other studies corroborate this conclusion, as the contributing factors to psychological health and quality of life are varied and numerous. For example, among the elderly, middle-aged, and young adults with or without MDD, high mindfulness levels positively corresponds to indicators of a good quality of life, such as emotional resilience, vitality, positive emotion, and physical health (42–46). It is important to note, however, that those with MDD have a lower quality of life compared to those without mental health issues. Even among euthymic patients with MDD, it has been shown that they have a reduced quality of life (47). Regardless, the determinants of general psychological health and quality of life independently and positively correspond to trait mindfulness among MDD patients.

This study revealed a significantly higher trait mindfulness level among married couples with MDD compared to their nonmarried counterparts. Being married can be the impetus for developing a higher level of mindfulness. Alternatively, a higher level of mindfulness corresponds to an increased chance of becoming married. Either way, emotional skills such as emotion recognition, communication, and anger regulation play important roles in the association between marital quality and mindfulness level (48). Happily married individuals with higher levels of mindfulness enjoy a higher quality of life. For example, during marital conflict, the level of cardiovascular reactivity of married couples inversely associates with their mindfulness levels (49). The level of satisfaction individuals have with their romantic relationships is positively related to their level of mindfulness (50). Furthermore, meditation promotes an individual’s acceptance of themselves and their partners, which corresponds to an improved marital wellbeing (51).

Although several studies have supported the positive association between marital status and mindfulness level, the underpinning mechanisms through which marital status regulates mindfulness level remains unclear. Our results reveal that being married positively corresponds with a higher performance on the observation facet of the FFMQ compared to those who are not married. The positive association between marital status and total mindfulness score is likely due to the factors that relate to a higher observation score on the FFMQ. For example, the FFMQ facet of nonjudgmental focus of attention on the present experience depends on the participation and regulation of observation. Therefore, we suggest that the regular and benign interactions of couples in marriage is conducive to the improvement of mindfulness level through the improvement of mindful observation.

Our results found that age independently and positively correspond with trait mindfulness level among MDD patients. Other studies have shown that nonclinical young adults have statistically greater anxiety, statistically greater experiential avoidance, and statistically lower mindfulness levels compared to nonclinical elders (52). There even appears to be a significant impact of age on benefit of mindfulness-based intervention to treat depression among adolescents (53). Age positively relates to the mindfulness aspects of present-moment attention, nonjudgment, acceptance, nonattachment, and decentralization. Nonjudgmental acceptance of present experience is an effective psychological adaptation strategy for aging individuals to reduce adverse emotional reactions caused by daily life stress (54, 55).

The association between trait mindfulness and MDD severity observed in our study might be at least in part explained by cultural influence. For example, most Chinese patients that have been newly diagnosed with cancer have low mindfulness levels and higher levels of depression (56, 57). However, it has been shown that German cancer patients have a low quality of life ranking and severe MDD, but they also have high levels of mindfulness. These results suggest that the relationship between depression and mindfulness level in the same disease state is complicated and affected by cultural factors (56, 57). Another study has shown that Western cultures compared to Eastern cultures have a stronger relationship between the trait mindfulness facet of nonjudgement and affective disorders (20). The same study revealed a stronger relationship between the trait mindfulness facet of description and affective disorders among Eastern cultures compared to Western cultures (20). Another recent study has revealed that the FMMQ suffers from conceptual and measurement shortcomings in a cross-cultural context (26). The influence of culture on mindfulness in patients with MDD warrants further investigation, especially as it relates to using the FFMQ as an assessment tool (58).

In conclusion, patients hospitalized for MDD present with various levels of trait mindfulness. Mindfulness-based therapeutic interventions have been shown to be beneficial in treating MDD. However, the efficiency of mindfulness-based therapeutic interventions is influenced by trait mindfulness level. Therefore, by monitoring trait mindfulness and identifying the independent factors that contribute to trait mindfulness, clinicians can identify MDD patients that would likely have a greater capacity to benefit from mindfulness-based therapy, more accurately establish a prognosis, and establish patient-specific mindfulness-related therapeutic targets. Furthermore, knowledge of a MDD patient’s baseline mindfulness level and their underlying factors can help ensure truly balanced clinical trial groups (59). Toward that end, we have identified that being married, being of older age, and having the perception of a good quality of life improves trait mindfulness levels via the five identified facets of mindfulness assessed by the FFMQ.

### Limitations

This study was based on analysis of inpatients with MDD from a single hospital during one calendar year. Although this ensures the consistency of inpatient screening, it also limits the generalizability of the results. Additionally, attempting to establish generalizable cross-cultural conclusions should be done with caution, since there is some evidence that the FFMQ is not sensitive to cultural differences. Additionally, since data was only collected at the point of hospital admission, the data used in this study is cross-sectional. In other words, the recorded patient’s mindfulness level and MDD characteristics might only reflect an ephemeral state. Therefore, it is impossible to deduce lasting causal relationships between the factors that are related to trait mindfulness. Furthermore, our current study cannot address the effect of different trait mindfulness levels on the outcome of mindfulness therapy. This will need to be addressed in a future, well-designed randomized clinical trial. Finally, the subjects involved in this study had MDD rather than mild-to-moderate depressive disorder. Further investigation on patients with mild-to-moderate depressive disorder is required to elucidate any generalizability of this study to these populations. Therefore, our results should be validated by well-designed prospective studies.

## Data availability statement

The raw data supporting the conclusions of this article will be made available by the authors, without undue reservation.

## Ethics statement

The studies involving human participants were reviewed and approved by Suzhou Guangji Hospital Ethics Committee. The patients/participants provided their written informed consent to participate in this study.

## Author contributions

G-HW, XD, RL, M-EN, and F-ZK conceptualized and designed the study. C-FJ, G-HW, L-LL, and F-ZK collected and analyzed the data. C-FJ, G-HW, M-EN, F-ZK, XD, and RL interpreted the data and drafted the manuscript. G-HW, XD, RL, and F-ZK revised the manuscript. All authors read and approved the final manuscript.

## Funding

F-ZK is funded by Top-Notch Talent Foundation (GSWS2021051), Suzhou Municipal Key Supporting Disciplines in Medicine (SZFCXK202103), Suzhou Clinical Medical Center for Mood Disorders (No. Szlcyxzx202109) from Suzhou Municipal Health Commission and Scientific Research Project (SZHL-A-202203) from Suzhou Municipal Nursing Association. G-HW is funded by Top-Notch Talent Foundation of Six Types of Outstanding Personnel from Jiangsu Provincial Health Commission (LGY2019015) and Top-Notch Talent Foundation of Suzhou Municipal Health Commission (GSWS2019057). The funders have no rule in conception, design, analysis, interpretation and writing of the manuscript.

## Conflict of interest

The authors declare that the research was conducted in the absence of any commercial or financial relationships that could be construed as a potential conflict of interest.

## Publisher’s note

All claims expressed in this article are solely those of the authors and do not necessarily represent those of their affiliated organizations, or those of the publisher, the editors and the reviewers. Any product that may be evaluated in this article, or claim that may be made by its manufacturer, is not guaranteed or endorsed by the publisher.
